# Study on Medication Rules of Traditional Chinese Medicine against Antineoplastic Drug-Induced Cardiotoxicity Based on Network Pharmacology and Data Mining

**DOI:** 10.1155/2020/7498525

**Published:** 2020-11-17

**Authors:** Wenchao Dan, Jinlei Liu, Xinyuan Guo, Boran Zhang, Yi Qu, Qingyong He

**Affiliations:** ^1^Department of Cardiology, China Academy of Chinese Medical Sciences Guanganmen Hospital, No. 5, North Line Pavilion, Xicheng District, Beijing 100053, China; ^2^Graduate School of Beijing University of Chinese Medicine, Beijing 100029, China; ^3^Department of Radiation Therapy, Cancer Hospital Chinese Academy of Medical Sciences, No. 17, South Panjiayuan, Chaoyang District, Beijing 100021, China

## Abstract

**Methods:**

The targets of antineoplastic drugs with cardiotoxicity were obtained from the National Center for Biotechnology Information (NCBI) database, China national knowledge infrastructure (CNKI) database, and Swiss Target Prediction platform. Then, the cardiotoxicity-related targets were derived from the Gene Cards, Disgenet, OMIM, and DrugBank databases, as well as the drug of current clinical guidelines. The targets both in these two sets were regarded as potential targets to alleviate ADIC. Then, candidate compounds and herbs were matched via Traditional Chinese Medicine Systems Pharmacology (TCMSP) platform. Cytoscape3.7.1 was used to set up the target-compound-herb network. Molecular docking between core targets and compounds was performed with AutodockVina1.1.2. The rules of herbs were summarized by analyzing their property, flavor, and channel tropism.

**Results:**

Twenty-one potential targets, 332 candidate compounds, and 400 kinds of herbs were obtained. Five core targets including potassium voltage-gated channel subfamily H member 2 (KCNH2), cyclin-dependent kinase 1 (CDK1), matrix metalloproteinase 2 (MMP2), mitogen-activated protein kinase1 (MAPK1), and tumor protein p53 (TP53) and 29 core compounds (beta-sitosterol, quercetin, kaempferol, etc.) were collected. Five core herbs (Yanhusuo, Gouteng, Huangbai, Lianqiao, and Gancao) were identified. Also, the TCM against ADIC were mainly bitter and acrid in taste, warm in property, and distributed to the liver and lung meridians.

**Conclusion:**

TCM against ADIC has great potential. Our study provides a new method and ideas for clinical applications of integrated Chinese and western medicine in treating ADIC.

## 1. Background

Since the 1970s, there have been reports of cardiotoxic reactions caused by anthracyclines [1]. With the continuous enrichment of cancer treatment methods, the survival time of cancer patients has been greatly improved, and there are more and more reports of cardiac toxicity. According to statistics, about 51% of cancer patients die from recurrent disease, and about 33% of them die from heart disease, which is related to the increased incidence of coronary artery disease and valve heart disease [2]. At present, there are many research studies related to cardiotoxicity in tumor treatment drugs. For example, tyrosine kinase (TK) is a ubiquitous phosphorylase that controls many signaling pathways, such as VEGF and EGFR. Inhibition of this pathway by antibodies has become a new method of clinical treatment, which is accompanied by damage to heart function after inhibition of signal transduction. For example, sunitinib can inhibit more than 50 different kinases to play an antitumor effect, but at the same time, inhibiting AMPK affects the oxidative stress response and increases the chance of heart failure [3]. ERBB receptor antibody is a commonly used drug in the clinical treatment of breast cancer. At the same time of treatment, it inhibits the signal transduction of neuromodulin 1 (NRG-1), affects the metabolism of cardiomyocytes, and leads to the development of cardiac toxicity [4]. Cardiotoxic reactions caused by anthracycline antibiotics are more common. During doxorubicin chemotherapy, reactive oxygen species (ROS) production, DNA damage, and mitochondrial dysfunction can be caused, resulting in increased left ventricular wall pressure and decreased left ventricular ejection fraction (LVEF), arrhythmia and highly symptomatic congestive heart failure, and other adverse consequences [5]. Radiation therapy is widely used in cancer treatment, and it also causes heart-related side effects during treatment. Its combination with anthracycline drugs also increases the risk of disease, which causes atherosclerosis, valve insufficiency, and other diseases [6].

At present, although the detection of certain biological markers can predict the impairment of cardiac function, such as troponin levels are associated with diastolic dysfunction caused by anthracyclines [7], the mechanism of most cardiac toxic drugs is still unclear. Only by using certain *β*-blockers, ACEI inhibitors, angiotensin receptor blockers, and other drugs for symptomatic treatment, the side effects of radiotherapy and chemotherapy can be alleviated [8]. The period of cardiotoxicity also has a great impact on tumor treatment. Early toxicity affects the progress of tumor treatment, and the tumor growth cannot be controlled in time, which leads to the deterioration of the condition; the late onset of toxicity often leads to the destruction of cardiac function, affecting the quality of life of patients. Moreover, the complexity of clinical symptoms increases the difficulty of distinguishing cardiac toxicity and cardiac dysfunction [9]. Therefore, an in-depth study of the mechanism of tumor cardiotoxicity can choose a more appropriate treatment approach for tumor patients and improve the quality of life of tumor patients.

TCM shows remarkable curative effects in clinical treatment of many diseases, and Chinese herbal medicine is a treasure trove of natural compounds, which have the advantages of wide source, multiple effective compounds, stable compound structure, and guaranteed safety. Reports have demonstrated that Xinmailong injection is effective for the treatment of cardiotoxicity caused by sequential chemotherapy of trastuzumab and anthracyclines in breast cancer, which could effectively improve the clinical symptoms of patients, attenuate myocardial injury, reduce blood viscosity, and inhibit expressions of serum IL-6 and TNF-*α* [10]. It was confirmed that Danshen injection has particular preventive effects on cardiotoxicity caused by chemotherapy of pyrroxine or epirubicin, and the effect includes protecting the myocardium, relieving myocardial damage, and improving cardiac function [11]. However, there is less research on the Chinese herbs medicine against cardiac toxicity caused by antineoplastic drugs. A meta-analysis study showed that Wenxin grenules can prevent and reduce tachycardia caused by anthracyclines, but its efficacy in improving overall efficiency and preventing and reducing atrial premature beats, ventricular premature beats, atrial fibrillation, and SOD levels is not clear; meanwhile, Baoxinkang can protect myocardial SOD activity [12]. Therefore, it is of great value to further explore Chinese herbs medicine against cardiac toxicity caused by antineoplastic drugs and to develop related preparations. However, TCM and compound medicines have the characteristics of multicompound, multitarget, and multimechanism system regulation. If a single drug, single target, and single research idea are used for research, it is difficult to systematically reflect the intervention mechanism of TCM; as an emerging research discipline, Chinese medicine started late in this field, and no research has systematically summarized the rules of TCM against cardiac toxicity caused by antineoplastic drugs.

Network pharmacology is a combination of system pharmacology, multidirectional pharmacology, bioinformatics, data mining, and other multidisciplinary. By constructing a network to analyze the relationship between the various compounds and the key nodes in the network, it could show the material basis and mechanism of action of Chinese medicine or compound [13, 14]. Therefore, it is currently widely used in the research of the mechanism of action and new drug development of Chinese medicine and compound.

**Figure 1:**
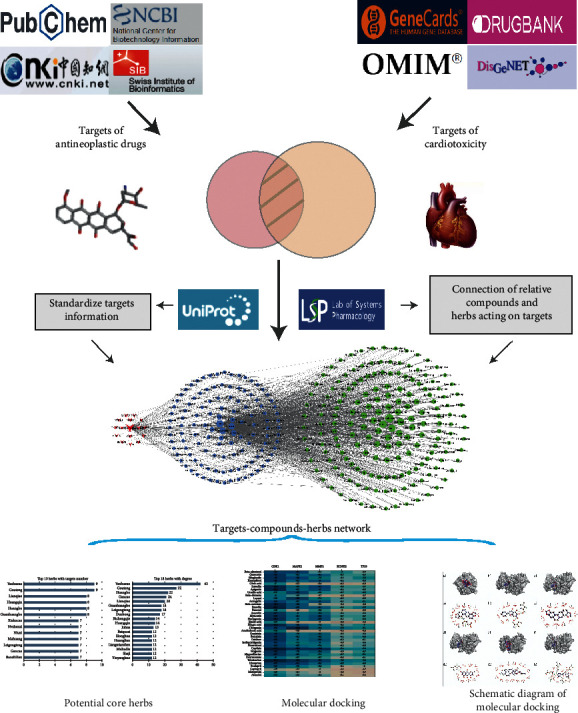
Whole framework based on network pharmacology.

In this study, we used the method of network pharmacology, used the relevant targets of cardiac toxicity caused by antineoplastic drugs as entry points, and matched the relevant ligands and TCM. As a result, we systematically predicted and analyzed the complex relationship between the target, compound and TCM, meanwhile initially discussed the material basis and general rules, which could provide ideas and theoretical basis for the follow-up selection of TCM, theoretical discussion, new drug development, and clinical integration of traditional Chinese and Western medicine. The flow chart of this study is shown in Figure 1.

## 2. Materials and Methods

### 2.1. The Collection of Potential Targets for Herbs against Antineoplastic Drug-Induced Cardiotoxicity

The ADIC is mainly manifested as arrhythmia, heart failure, and ischemic heart disease [15]. To obtain relevant targets, cardiotoxicity-related words, such as “arrhythmia,” “heart failure,” and “myocardial ischemia,” were used as keywords to query the following electronic databases: Genecards database [16] (https://www.genecards.org/), OMIM database [17] (https://omim.org/), DrugBank database [18], and Disgenet database [19] (http://www.disgenet.org). Results were merged, and duplicates were removed to obtain target group I. The sdf format files of antineoplastic drugs, including anthracyclines (doxorubicin (DOX), epirubicin, pirarubicin, aclarubicin, idarubicin, amrubicin, and daunorubicin), taxanes (paclitaxel and docetaxel), and tyrosinase inhibitors, were derived from Pubchem, and used to predict compound targets via the SwissTargetPredicition platform [20] (http://www.swisstargetprediction.ch/). Then, we supplemented the results by consulting the literature about the targets related to human epidermal growth factor receptor 2 (HER-2) inhibitors and immune checkpoint blockade therapy. After removing the duplicate targets, we obtained target group II. The recommended drugs for ADIC were confirmed by consulting guidelines and consensus [21–23], and their targets were set as target group III. Then, the intersection of target group I and II were merged with target group III to get the final dataset of potential targets for TCM against ADIC. The information of these targets was standardized using the Uniprot database [24] (https://www.uniprot.org/).

### 2.2. Candidate Compound Screening and Target-Compound Network Construction

By searching the Traditional Chinese Medicine Systems Pharmacology (TCMSP) platform [25] (http://tcmspw.com/tcmsp.php), the compounds that can act on relevant targets are collected. According to the pharmacokinetic parameters (ADME) and the Lipinski rules [26], candidate compounds were screened. The specific screening criteria were oral availability (OB) ≥ 30%, drug likeness [27] (DL) ≥ 0.18, drug half-life > 4 h, topological polar surface area [28] < 140 angstroms, rotatable bonds number [29] ≤ 10; and molecular weight ≤ 500 Da. Considering that the parameters provided by the TCMSP platform is predicted by the computer, part of the data may be inconsistent with the actual situation, and the deleted compounds were checked one by one to supplement the relevant active compounds after the initial screening. The revised compounds were regarded as candidate compounds. Along with these compounds, the potential targets were imported into Cytoscape3.7.2 software [30] to construct a target-compound network and calculate the topology parameter (degree) of the nodes in the network. Core targets, compounds, and herbs were defined based on the degree.

### 2.3. Herb Matching and Target-Compound-Herb Network Construction

We collected herbs containing candidate compounds and constructed the compound-herb network. The “target-compound” network and the compound-herb network were combined through the merge function module in the Cytoscape3.7.2 to establish the target-compound-herb network. Furthermore, we identified key nodes based on the degree of each node in the network and evaluated the strength of efficacy of the Chinese medicines and compounds in treating ADIC.

### 2.4. Characteristics of the Candidate Herbs

A frequency analysis was performed to summarize the rules of candidate herbs against ADIC. Characteristics under investigation included property, flavor, and channel tropism. Considering the heterogeneity of the information on characteristics from different sources, the priority order of standards we adopted in this study was *Chinese Pharmacopoeia* (2015) [31], *Chinese Pharmacy* (“Thirteenth Five-Year Plan” textbook) [32], and *Chinese Dictionary of Clinical Medicine* [33]. Herbs lacking of relevant information were deleted.

### 2.5. Molecular Docking of the Target-Compound

To assess the credibility of the connection between the target and the compound and identify new candidate herbs for ADIC treatment, molecular docking of the core compounds with core targets was carried out. Five targets with the highest degree in the “target-compound-herb” network were treated as receptors, and core compounds, as well as anti-ADIC drugs (atenolol, captopril, dexrazoxane, enalapril, irbesartan, metoprolol, and telmisartan) recommended by the guideline, were treated as ligands.

The crystal structure of the 5 proteins was downloaded from the Protein Data Bank (http://www.rcsb.org/pdb) and saved in a PDB format. The three-dimensional (3D) conformer structure of candidate compounds was downloaded from the PubChem database (https://pubchem.ncbi.nlm.nih.gov/) and saved in a SDF format that was subsequently converted to a PDB format with Open babel 2.4.1. Ligands and receptors were prepared via AutoDock Tools (v.1.5.6) and PyMOL (v.2.3). The work of preparing receptors included deleting the original ligands and water molecules from the crystal structure of receptors, adding nonpolar hydrogens, and calculating Gasteiger partial charges. The flow of handling ligands contained applying energy minimization and assigning atomic charges and atom. All the prepared receptors and ligands were saved in a pdbqt format.

Then, the affinity indicating binding strength between the ligand and the target protein was evaluated with Autodock Vina (v.1.1.2). Affinity < −4.25 kcal/mol means that ligands and receptors have possibility of combination, affinity < −5.00 kcal/mol indicates good binding strength, and affinity < −7.00 kcal/mol suggests satisfactory binding strength [34]. Pymol 2.3 and LigPlot 2.2 were used to visualize and analyze the docked conformations, and from the binding conformations of the docking results of each compound, the docking results with lower binding energy and better conformation were selected for presentation.

## 3. Results

### 3.1. Target Acquisition and Screening

A total of 84 antineoplastic drug-induced cardiotoxicity targets were obtained through Swisstarget Prediction and the literature, and 2,059 cardiotoxicity-related targets were obtained through the disease databases. A total of 40 targets were obtained from the intersection of the aforementioned two datasets. Additional 4 targets (ACE, AGTR1, AGTR2, and IFNAR1) of the anti-ADIC drugs (ACEI, ARB, and beta-blockers) recommended by clinical guidelines were also counted. Among these 44 targets, 21 were successfully matched to compounds that met the ADME and Lipinski screening criteria and were defined as potential targets for herbs against ADIC, as shown in Table 1.

### 3.2. Candidate Compound Acquisition and Target-Compound Network Construction

**Figure 2:**
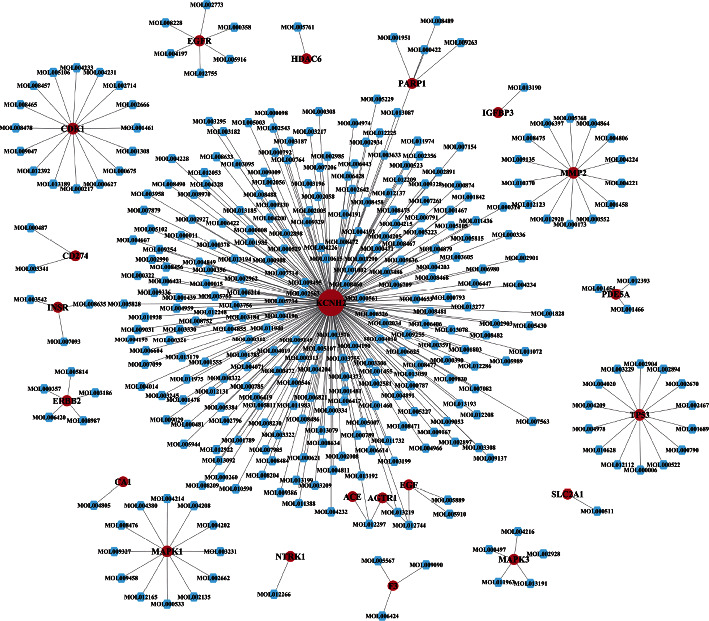
Target-compound network.

The potential targets were mapped by 13,729 small-molecule compounds in the TCMSP database, among which 332 candidate compounds met the ADME and Lipinski criteria. The target-compound network constructed by the potential targets and candidate compounds is shown in Figure 2. The network consisted of 353 nodes and 337 edges. The red nodes in the figure represent target points, the blue nodes represent compounds, the edges represent relations between two adjacent nodes, and the degrees represent the number of connected edges of nodes. The larger the degrees are, the greater regulatory roles the nodes play in the entire network. Figure 2 shows that KCNH2, CDK1, MMP2, MAPK1, and TP53 were the core targets, and their corresponding degrees were 239, 16, 15, 14, and 12, respectively. Single targets with multiple compounds dominated the network but not vice versa.

### 3.3. Herb Acquisition and Target-Compound-Herb Network Construction

**Figure 3:**
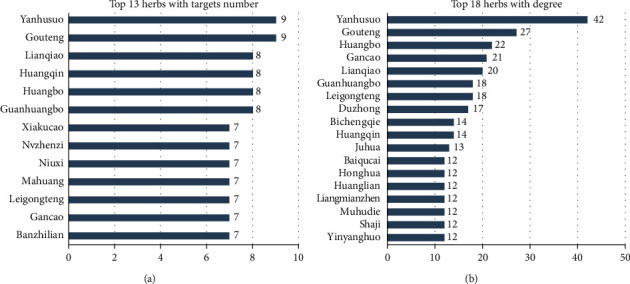
Distribution of candidate compounds and target sites in herbs.

A total of 446 herbs were matched to the 332 compounds through the TCMSP database, 46 of which were not included in the *Chinese Pharmacopoeia* (2015), *Chinese Pharmacy* (“*Thirteenth Five-Year Plan” textbook*), and *Chinese Dictionary of Clinical Medicine*, so we finally collected 400 herbs. Based on this, we constructed a compound-herb network. The network contained 732 nodes and 1,925 edges. Among them, the top 8 herb nodes with the highest degree were Yanhusuo, Gouteng, Huangbo, Gancao, Lianqiao, Guanhuangbo, Leigongteng, and Duzhong, and they contained 42, 27, 22, 21, 20, 18, 18, and 17 candidate compounds, respectively. The targets of herbs were collected through the bridging of candidate compounds. The results showed that Yanhusuo, Gouteng, Lianqiao, Huangqin, Huangbo, Guanhuangbo, Xiakucao, and Nvzhenzi contained the most targets (9, 9, 9, 9, 8, 8, 8, and 8, respectively). Therefore, we speculate that the abovementioned herbs have stronger regulatory effect on alleviating ADIC in the 400 herbs. The number of candidate compounds and targets of the herbs is shown in Figure 3. The median of the compound degree was 4, and compounds with a degree greater than 8 were considered as potential core compounds. The top 5 compounds were beta-sitosterol, quercetin, daucosterol, kaempferol, and oleic acid. The remaining compounds are shown in Table 2.

**Figure 4:**
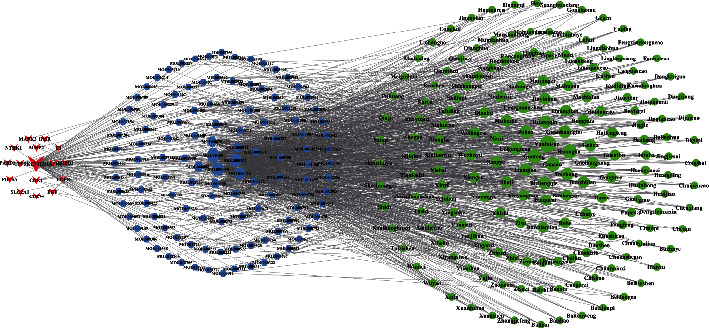
Target-compound-herb network (degree of herb ≥5).

In order to present the relation among potential targets, compounds, and herbs more concisely, our study reconstructed the target-compound-herb network by selecting herbs with degree **≥**5 and their related compounds and targets, as shown in Figure 4. In the figure, the red nodes represented targets, the blue nodes represented compounds, and the green nodes represented herbs. The size of each node was positively correlated with the degree.

### 3.4. Properties, Tastes, and Meridian Tropism of Herbs

**Figure 5:**
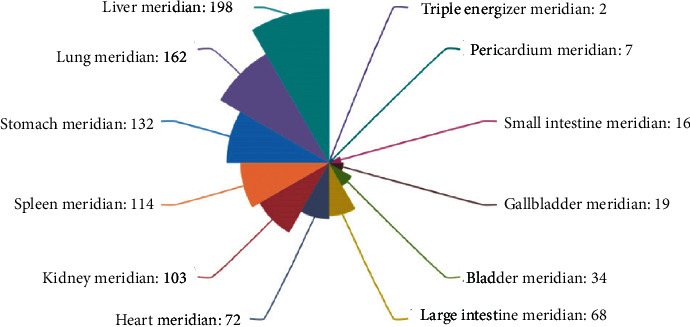
Meridian tropism of herbs.

By analyzing the information of properties, tastes, and meridian tropism of the 400 herbs intervening ADIC, our results showed that herbs with bitter taste had the highest frequency, followed by acrid and sweet taste, and the proportion of the three tastes was 80.51%; most herbs were warm. In terms of the meridian tropism, herbs belonging to liver meridian tropism accounted for the most proportion among the 400 herbs, which was 21.36%, herbs belonging to lung meridian tropism accounted for 17.48%, and herbs belonging to both liver and lung meridian tropism accounted for 38.83%. The results are shown in Table 3 and Figure 5.

### 3.5. Molecular Docking

Twenty-nine core potential compounds were molecularly docked with 5 core targets including KCNH2 (PDBID: 6SYG), CDK1 (PDBID: 4Y72), TP53 (PDBID: 3Q05), MMP2 (PDBID: 1RTG), and MAPK1 (PDBID: 6SLG). A total of 145 pairs of receptor-ligand combinations were obtained, among which 79 combinations had affinity < −7 kcal/mol, accounting for 54.48%. Twenty-five combinations were in the target-compound network, with the strongest binding in MMP2-coptisine (−10.2 kcal/mol) and the weakest in KCNH2-arachidonic acid (−4.4 kcal/mol). The average value of the 25 combinations was −6.684 kcal/mol, suggesting that the binding between the potential core targets and the compounds was strong.

**Figure 6:**
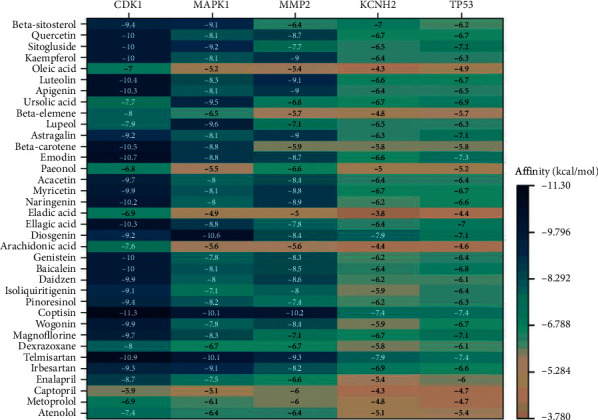
Results of molecular docking.

The remaining 120 combinations were outside the target-compound network. The top 3 combinations with the highest affinity were CDK1-coptisine (−11.3 kcal/mol), CDK1-emodin (−10.7 kcal/mol), and MAPK1-diosgenin (−10.6 kcal/mol). There were 6 combinations with affinity < −10.2 kcal/mol. The docking strength of these 6 combinations exceeded that of the 25 combinations in the target-compound network. The docking results indicated that the number of potential compound-target combinations may be far greater than that of the compound-target combinations included in the TCMSP database, which shows that there are still a large number of connections between active TCM compounds and cardiotoxicity targets waiting to be further studied and excavated. The docking results can provide data support for future experimental screening of related TCM and compounds. The results are shown in Figure 6.

**Figure 7:**
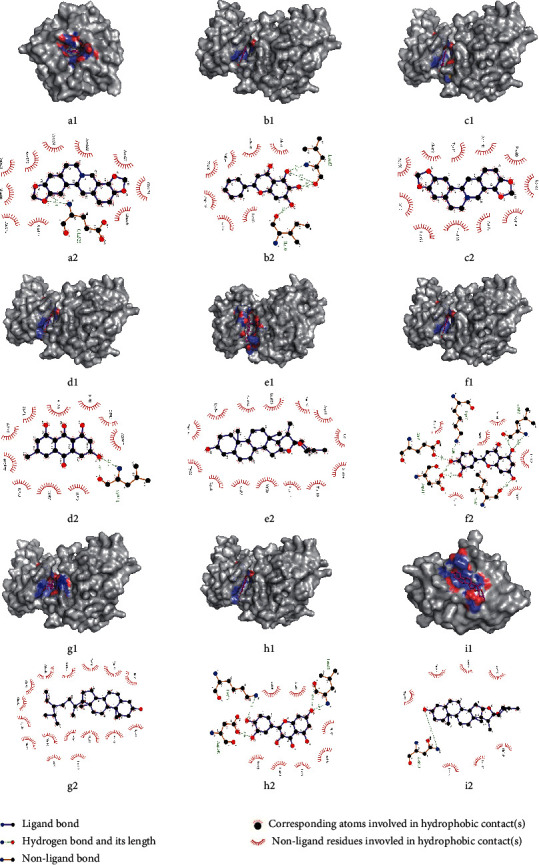
Molecular docking of compounds with core targets. (a) MMP2-coptsine; (b) CDK1-baicalein; (c) CDK1-coptsine; (d) CDK1-emodin; (e) MAPK1-diosgenin; (f) CDK1-luteolin; (g) CDK1-*β*-sitosterol; (h) CDK1- quercetin; (i) KCNH2-diosgenin.

Comprehensively considering the molecular docking affinity value and the degree value in the target-compound-herb network, the results of docking with affinity < −10 kcal/mol in the network and those with the top 4 affinity outside the network were shown in the 3D and 2D format. It can be seen from the figure that each compound ligand is embedded in the active pocket of the receptor target and interacts with multiple residues of the target through hydrophobic interaction and hydrogen bond. The diagrams are shown in Figures 7(a)∼7(h).

## 4. Discussion

A number of reports have shown the potential of natural herbs in decreasing the cardiotoxic effects of chemotherapeutic agents on healthy cells, without negatively affecting their antineoplastic activity [35]. Subsequent improvement in heart function and quality of life may make chemotherapy more sustainable and reduce treatment-related mortality. Besides, most of TCM has anticancer effects with few side effects. Some physicians, thus, turned to use TCM as an alternative or complementary treatment to prevent and treat ADIC [36]. However, systematic studies of TCM intervention on ADIC are still insufficient. It is of great clinical significance to study the intervention mechanism of TCM against ADIC and to develop related pharmaceutical preparations on this basis.

### 4.1. Targets

In this study, 5 core targets (KCNH2, CDK1, MMP2, MAPK1, and TP53) were identified from the target-compound-herb network. Dysfunction of KCNH2 activation will lead to the decrease of hERG current and a prolongation of the action potentials recorded in cardiomyocytes [37]. Dysfunction of KCNH2 expression is related to QT prolongation syndrome [38]. Inhibition of the KCNH2 potassium channel can lead to cardiotoxicity [39].

The overexpression of four regulatory factors of cell cycle including CDK1 was shown to efficiently induce cell division in postmitotic mouse, rat, and human cardiomyocytes, lead to a significant improvement in cardiac function after acute or subacute myocardial infarction, and improve heart function as indicated by significant improvement in the ejection fraction, stroke volume, and cardiac output.

MMP-2 can hydrolyze sarcomeric proteins in the heart, remodel the extracellular matrix, and lead to cardiotoxicity. Anthracycline drugs such as DOX can enhance the activity of MMP-2 in cardiomyocytes and may cause heart failure, so inhibition of MMP-2 activity can interfere with the cardiotoxicity of anticancer drugs [40].

DOX-induced mitochondrial reactive oxygen species (ROS) release was demonstrated to activate ERK-mediated heat shock factor 2 (HSF2) nuclear translocation and AT1 R upregulation, which caused DOX-damaged heart failure [41]. It is suggested that the activation of the ERK2 pathway of the mitogen-activated protein kinase (MAPK) family can protect cardiomyocytes from DOX-induced cardiotoxicity [42].

The wild-type p53 protein, which is encoded by the TP53 gene, plays an important role as a tumor suppressor in regulation of cell cycle arrest, DNA repair, and apoptosis. The association between p53 overexpression, mutations, and drug resistance has been reported in bladder, breast, ovarian, and other types of cancer. Mutation of p53 protein was indicated to decrease sensitivity to anthracycline treatments in human bladder TCC cells [43]. Reactivation of mutant p53 in those tumor cells may restore p53 tumor-suppressor function and sensitize mt-p53 cells to chemotherapy treatments [44]. Therefore, this study speculates that regulation of TP53 can enhance the sensitivity of tumor to anthracycline and other chemotherapy drugs, so as to reduce the dosage of these drugs and decrease their cardiotoxicity accordingly.

### 4.2. Compound

The core compounds obtained in this study included beta-sitosterol, quercetin, kaempferol, and oleic acid. Studies have shown that one of the core mechanisms of cardiotoxicity caused by anthracyclines is that ROS and reactive nitrogen species (RNS) will accumulate along with the metabolism of anthracyclines. A large amount of ROS and RNS may activate cytotoxic signals [45] in cardiomyocytes, leading to cardiac dysfunction through DNA damage, mitochondrial dysfunction, decreased antioxidant enzymes, and intracellular calcium homeostasis [46].

The intake of beta-sitosterol can enhance the storage of nonenzymatic antioxidants such as glutathione 1, vitamin C, and vitamin E [47]. Glutathione, the main antioxidative stress buffer in the cell, is widely used in cancer treatment. It mainly exists in the form of reduced glutathione (GSH) and oxidized glutathione (GSSG) in the human body. Experiments have shown that beta-sitosterol has a protective effect on the reduction of antioxidants containing GSH in colon and liver tissues induced by 1, 2-dimethyl hydrazine [48]. Therefore, it is speculated that beta-sitosterol may mitigate ADIC by increasing the content of nonenzymatic antioxidants to compensate for the absence of antioxidant enzymes caused by anthracyclines.

Research showed that quercetin had significant anticancer activities against MCF-7, SKBR-3, and MDA-MB-231 human breast cancer cells and CT26 mouse colon cancer cells. It could also reduce the cardiotoxicity caused by DOX in the Balb/*c* mice model [49].

Some studies demonstrated that kaempferol protected against DOX-induced cardiotoxicity by regulating the 14-3-3*γ*, MAPK, and ADMA/DDAHII/eNOS/NO pathway, inhibiting the activation of p53-mediated, mitochondrion-dependent apoptotic signaling, and improving mitochondrial function [50]. It has been reported that kaempferol dose dependently restored hemodynamic, left ventricular functions, decreased cardiac injury marker enzymes in serum, increased antioxidant levels, and reduced lipid peroxidation, TNF-*α* level, and apoptosis, so to alleviate cardiotoxicity [51].

Oleic acid has been found to have an excellent antioxidant effect. It was shown that oleic acid had a protective effect on myocardial mitochondrial injury induced by adrenaline in several aspects, such as inhibiting the elevated levels of lipid peroxidation and mitochondrial DNA damage, maintaining membrane intactness and the level of glutathione and keeping mitochondrial enzyme and membrane potential stable in rat experiments [52].

In conclusion, the potential core targets and compounds screened in this study were related to cardiotoxicity, which may play an important protective role in exploring the effects of TCM on ADIC.

### 4.3. Molecular Docking

The results of molecular docking showed that, in the target-compound network, the combination of MMP2-coptisine performed best, with affinity of −10.2 kcal/mol. There is considerable accumulating evidence suggesting a role of MMP-2, MMP-3, MMP-9 in promoting tumor cell invasion and infiltration [53,54]. The activation of the PI3K/Akt pathway associated with matrix metalloproteinases (MMPs) is essential for the growth and survival of cancer cells. Coptisine is a natural isoquinoline alkaloid. Studies have confirmed that coptisine has an improvement effect on cardiovascular disease in vitro and in vivo. It has strong antioxidant activity, helps maintain cell membrane integrity, improves mitochondrial respiratory dysfunction, and reduces cardiomyocyte apoptosis [55]. At the same time, it can protect cardiomyocytes from hypoxia or reoxidation-induced damage by inhibiting autophagy [56]. Simultaneously, coptisine can intervene in cancer through multiple ways. For instance, coptisine was found to induce apoptosis by strengthening the expression of the 67kD laminin receptor/cGMP pathway in various human hepatoma cells [57]. It can also affect a variety of apoptosis-related targets, including Apaf-a, ROS, Bcl-2-XL, Bax, cytochrome c, Bid, AIF, XIAP, caspase-3, and caspase-9 to inhibit the activity, adhesion, and migration of HCT-116 cells [58]. The abovementioned studies proved the reliability of this target-compound combination relationship in the network. We speculate that if coptisine is developed as a clinical drug, it may reduce the dose of cardiotoxic antitumor drugs, and at the same time, it may alleviate the oxidative stress response in cardiomyocytes caused by antitumor drugs, thereby improving myocardial function.

In addition to the target-compound network, the molecular docking results also showed that the six combinations of CDK1-coptisine, CDK1-emodin, MAPK1-diosgenin, CDK1-luteolin, CDK1-Apigenin, and CDK1-ellagicacid had good binding activity. Among them, emodin has been widely used in clinics because of its various pharmacological properties such as anticancer, antibacterial, antiviral, and liver protection properties [59]. Luteolin has been used clinically as a natural flavonoid. Clinical trials have shown that preparations containing luteolin have good anti-inflammatory effects [60], and in the treatment of glioma, combined with olaparib (PARP inhibition agent) or ionizing radiation treatment has certain clinical value [61]. Therefore, it can be regarded as one of the candidate drugs dealing with ADIC in future experimental or clinical researches.

### 4.4. Herbs

In order to better cooperate with tumor drug treatment, the selected herb can not only protect the heart but also has a certain antitumor effect. The core herbs obtained in this study included Yanhusuo, Gouteng, Huangbai, Lianqiao, and Gancao. Yanhusuo has the most therapeutic potential. Modern pharmacological research showed that corydalis had many functions such as antiarrhythmia, antimyocardial infarction, coronary artery expansion, antitumor, and antithrombosis functions [62], and the extract from corydalis had a protective effect on myocardial ischemia-reperfusion injury, which was closely associated with the inhibition of myocardial apoptosis through modulation of the Bcl-2 family [63]. Tetrahydropalmatine (THP), an effective compound of corydalis, with the analgesic effects on bone cancer pain may underlie the inhibition of microglial cell activation and the increase in proinflammatory cytokine. Meanwhile, THP can reverse the ventricular tachycardia induced by drugs in rabbits and exert an antiarrhythmia effect [64].

Alkaloid extracted from Gouteng may intervene in tachycardia and arrhythmia through blocking the calcium channel and opening the potassium channel [65]. Isorhynchophylline, extracted from Gouteng, has the potential of inhibiting cardiac hypertrophy through Nrf2 nuclear translocation and the MAPK pathway [66].

Huangbai has showcased a wide range of pharmacological effects, including anticancer, anti-inflammatory, and antimicrobial effects. The compound ((21S, 23R) epoxy-24-hydroxy-21*β*, 25-diethoxy) tirucalla-7-en-3-one has a relatively strong effect as adriamycin against four tumors. Polysaccharides from an aqueous extract of phellodendri act on cell-mediated stimulation and humoral immunity instead of tumor cell inhibition to exert tumoricidal activity [67]. Meanwhile, Huangbai has an antiarrhythmic effect [68].

The main components of Lianqiao suspense are phillyrin, forsythiaside, chlorogenic acid, and so on. Phillyrin can improve heart function by regulating the myocardial contractility index and reduce myocardial fibrosis after myocardial infarction, which showed an effect similar to that of enalapril [69]. Forsythiaside can effectively prevent hydrogen peroxide-induced mitochondrial membrane potential depolarization. Cytochrome *C*, caspase-9, and caspase-3 are markers of mitochondrial-dependent apoptotic pathways that induce cell death, which is an oxidative stress reaction. Forsythiaside can effectively prevent this reaction induced by hydrogen peroxide [70]. Chlorogenic acid (CGA) attenuates TNF-*α*-induced cardiomyocyte apoptosis by inhibiting JNK [71]. Li et al. found that CGA could inhibit isotype-induced cardiac hypertrophy through blocking the NF-*κ*B signaling pathway [72]. CGA can significantly reverse the decline in cardiac mitochondrial membrane potential induced by TNF-*α*. Therefore, it is suggested that inhibiting TNF-*α* may help maintain the stability of mitochondrial membrane potential and prevent adriamycin-induced cardiotoxicity in isolated rat hearts [73].

Gancao has protective effects on the heart, and the hydrolysates of its main components glycyrrhizin is glycyrrhetinic acid, which has the effect of intervening the permeability transition (MPT) of rat heart mitochondria [74]. The extract of Gancao can maintain the integrity of the cell membrane, improve lipid homeostasis, and stabilize actin. Gancao extract can reduce ROS levels and repair antioxidant status, thus helping to repair DNA damage and mitochondrial function. The SIRT-1-mediated pathway and its downstream activator PPARS are important for the maintenance of cell function. Glycyrrhiza extract could restore nuclear SIRT-1 and PPAR-*γ* levels in H9C2 cardiomyocytes reduced by DOX interference [75]. It is concluded that Gancao extract can antagonize the cardiotoxicity induced by DOX and maintain the normal function of cardiac myocytes.

The herbs mentioned above may have the potential of mitigating ADIC; meanwhile, some of them have extra beneficial effects on cancer patients. This will provide an alternative scheme for related trials on treating ADIC with TCM. At the same time, the information of herbal taste and meridian tropism can lay a certain foundation for the theoretical discussion and research of TCM on ADIC in the future. Herbs with multiple targets might be of great importance due to their potential broad-spectrum effects against ADIC. A combination of herbs containing various compounds corresponding to multiple targets might be an alternative strategy. The obtained core herbs and compounds can provide reference for the prescription exploration in integrating traditional Chinese and Western medicine to treat ADIC.

## 5. Strengths and Limitations

The advantage of this study lies in the use of network pharmacology, data mining, and molecular docking methods to obtain herbs and compounds that can potentially resist ADIC from a large number of TCM, which can save economic cost and time in the future study. Meantime, many rare diseases still lack effective means, and the efficacy of some common diseases is not ideal. Chinese medicine may have certain advantages, but screening out the ideal herb is very time consuming and costly, such as artemisinin. The method used in this research can provide a reference for similar research to narrow the selection scope. The deficiency of this study is that we focus on the relation among targets, compounds, and herbs, but the efficacy of herbs between the targets and the compounds and the compound content of each herb have not been fully discussed. There is a wide variety of antitumor drugs, and new drugs are emerging one after another. The drugs selected in this study came from the existing literature. However, some new drugs with potential cardiotoxicity are not included because of insufficient clinical evidence and no relevant reports have been seen so far. Thus, we will continue to follow-up related progress in the future.

## 6. Conclusions

This study constructed a target-compound-herb network using ADIC-related targets from multiple databases and compounds and herbs matched from the TCMSP platform. Core targets (KCNH2, CDK1, and MMP2), compounds (beta-sitosterol, quercetin, and kaempferol), and herbs (Yanhusuo, Gouteng, Huangbai, Lianqiao, and Gancao) that may be helpful in treating ADIC were identified from the network. The binding activity between the core targets and the core compounds were strong, verifying the reliability of their connection beyond the network. Most of the candidate herbs were bitter, acrid, and warm, belonging to liver and lung meridians. These conclusions provide new ideas for the screening of herbs that can intervene ADIC in an advanced, evidence-based, and systematic way, and also provide reference for the prescription combining TCM and Western medicine.

## Figures and Tables

**Table 1:** Information of potential targets.

ID	Gene symbol	Uniprot ID	Protein name
1	ACE	P12821	Angiotensin-converting enzyme
2	AGTR1	P30556	Type-1 angiotensin II receptor
3	CA1	P00915	Carbonic anhydrase I
4	CD274	Q9NZQ7	Programmed cell death 1 ligand 1
5	CDK1	P06493	Cell division control protein 2 homolog
6	EGF	P01133	Proepidermal growth factor
7	EGFR	P00533	Epidermal growth factor receptor
8	ERBB2	P04626	Receptor tyrosine-protein kinase erbB-2
9	F3	P13726	Tissue factor
10	HDAC6	Q9UBN7	Histone deacetylase 6
11	IGFBP3	P17936	Insulin-like growth factor-binding protein 3
12	INSR	P06213	Insulin receptor
13	KCNH2	Q12809	Potassium voltage-gated channel subfamily H member 2
14	MAPK1	P28482	Mitogen-activated protein kinase 1
15	MAPK3	P27361	Mitogen-activated protein kinase 3
16	MMP2	P08253	72 kDa type IV collagenase
17	NTRK1	P04629	High affinity nerve growth factor receptor
18	PARP1	P09874	Poly [ADP-ribose] polymerase 1
19	PDE5A	O76074	CGMP-specific 3′, 5′-cyclic phosphodiesterase
20	SLC2A1	P11166	Solute carrier family 2, facilitated glucose transporter member 1
21	TP53	P04637	Cellular tumor antigen p53

**Table 2:** Information of candidate core compounds (degree > 8).

MolID	MolName	CAS	Degree	OB	DL
MOL000358	Beta-sitosterol	83-46-5	225	36.91391	0.75123
MOL000098	Quercetin	117-39-5	174	46.43335	0.27525
MOL000357	Sitogluside	474-58-8	170	20.63194	0.6241
MOL000422	Kaempferol	520-18-3	122	41.88225	0.24066
MOL000675	Oleic acid	112-80-1	109	33.12836	0.14243
MOL000006	Luteolin	491-70-3	83	36.16263	0.24552
MOL000008	Apigenin	520-36-5	75	23.06216	0.21306
MOL000511	Ursolic acid	77-52-1	72	16.7749	0.75457
MOL000908	Beta-elemene	515-13-9	45	25.63362	0.060519
MOL000356	Lupeol	545-47-1	36	12.12076	0.77716
MOL000561	Astragalin	480-10-4	35	14.02685	0.73616
MOL002773	Beta-carotene	7235-40-7	28	37.18433	0.58358
MOL000472	Emodin	518-82-1	28	24.39832	0.23916
MOL000874	Paeonol	552-41-0	26	28.78724	0.039185
MOL001689	Acacetin	480-44-4	20	34.97357	0.24082
MOL002008	Myricetin	529-44-2	20	13.74833	0.31057
MOL004328	Naringenin	67604-48-2	20	59.2939	0.21128
MOL001308	Elaidic acid	112-79-8	17	33.12836	0.14262
MOL001002	Ellagic acid	476-66-4	17	43.06456	0.43417
MOL000546	Eiosgenin	512-04-9	14	80.87792	0.80979
MOL001439	Arachidonic acid	506-32-1	13	45.57325	0.20409
MOL000481	Genistein	446-72-0	13	17.93288	0.21384
MOL002714	Baicalein	491-67-8	12	33.51892	0.20888
MOL000390	Daidzein	486-66-8	11	19.44106	0.18694
MOL001789	Isoliquiritigenin	961-29-5	11	85.3218	0.14805
MOL001842	Pinoresinol	487-36-5	11	4.250519	0.5159
MOL001458	Coptisine	3486-66-6	10	30.67185	0.85647
MOL000173	Wogonin	632-85-9	10	30.68457	0.22942
MOL002891	Magnoflorine	2141/9/5	9	0.479985	0.54735

**Table 3:** Properties and tastes of herbs.

Taste	Frequency	Proportion (%)	Nature	Frequency	Proportion (%)
Bitter	222	35.58	Warm	107	33.23
Acrid	165	26.44	Cold	97	30.12
Sweet	150	24.04	Slight cold	48	14.91
Astringent	31	4.97	Cool	36	11.18
Sour	29	4.65	Slight warm	24	7.45
Salty	16	2.56	Hot	10	3.11
Light	11	1.76			

## Data Availability

The datasets used and/or analyzed during the current study are available from the corresponding author on reasonable request.

## Abbreviations

TCM: Traditional Chinese medicine
TCMSP: Traditional Chinese Medicine Systems Pharmacology
DL: Drug likeness
OB: Oral availability
HL: Drug half-life
TPSA: Topological polar surface area
RBN: Rotatable bonds number
MW: Molecular weight
PDB: Protein Data Bank
3D: Three dimensional
KCNH2: Potassium voltage-gated channel subfamily *H* member 2
CDK1: Cyclin-dependent kinase 1
MMP2: Matrix metalloproteinase 2
MAPK1: Mitogen-activated protein kinase1
MAPK: Mitogen-activated protein kinase
TP53: Tumor protein p53
TK: Tyrosine kinase
VEGF: Vascular endothelial growth factor
EGFR: Epidermal growth factor receptor
AMPK: Adenosine 5′-monophosphate- (AMP-) activated protein kinase
ERBB2: Human epidermal growth factor receptor (HER-2)
NRG-1: Neuromodulin 1
ROS: Reactive oxygen species
LVEF: Left ventricular ejection fraction
ACEI: Angiotensin-converting enzyme inhibitor
SOD: Superoxide dismutase
ARB: Angiotensin receptor blocker
hERG: Human Ether-a-go-go Related Gene
EF: Ejection fraction
ERK1/2: Extracellular regulatory protein kinase1/2
AT1 R: Angiotensin receptor1
DOX: Doxorubicin
TCC cells: Transitional cell carcinoma
mt-p53: Mutation of p53
RNS: Reactive nitrogen species
GSH: Glutathione
GSSG: Oxidized glutathione
DMH: 1: 2-dimethy lhydrazine
TNF-*α*: Tumor necrosis factor-*α*
ADMA: Asymmetric dimethylarginine
DDAH II: Dimethylarginine dimethylamine hydrolase II
eNOS: Endothelial nitric oxide synthase
NO: Nitric oxide
MCF-7: Human breast cancer cells MCF-7
SKBR-3: Human breast cancer cells SKBR-3
MDA-MB-231: Human breast cancer cells MDA-MB-231
CT26: Mouse colon cancer cells CT26
FoxO1: Forkhead box O1
NFATc3: Nuclear factor of activated T-cells, cytoplasmic, calcineurindependent 3
PI3K/Akt: Phosphatidylinositol 3 kinase / protein kinase B
MMPs: Matrix metalloproteinases
cGMP: Cyclic guanosine monophosphate
Apaf-a: Apoptotic protease activating factor-a
Bax: BCL2-associated X
BID: BH3-interacting domain death agonist
AIF: Apoptosis inducing factor
XIAP: x-linked inhibitor of apoptosis protein
HCT-116: Human colon cancer Cell HCT-116
PARP: Poly ADP-ribose Polymerase
IR: Ionizing radiation
Bcl-2: b-cell lymphoma-2
THP: Tetrahydropalmatine
VT: Ventricular tachycardia
Nrf2: Nuclear factor E2-related factor 2
CGA: Chlorogenic acid
